# Draft Genome Sequences of Two Xanthomonas euvesicatoria Strains from the Balkan Peninsula

**DOI:** 10.1128/genomeA.01528-14

**Published:** 2015-02-05

**Authors:** Taca Vancheva, Pierre Lefeuvre, Nevena Bogatzevska, Penka Moncheva, Ralf Koebnik

**Affiliations:** a Faculty of Biology, Sofia University St. Kliment Ohridski, Sofia, Bulgaria; b UMR 186 IRD-Cirad-Université Montpellier 2 Résistance des Plantes aux Bioagresseurs, Montpellier, France; c CIRAD, UMR PVBMT, Pôle de Protection des Plantes, Saint-Pierre, Ile de la Réunion, France; d Institute of Soil Science, Agrotechnologies and Plant Protection Nikola Poushkarov, Sofia, Bulgaria

## Abstract

We report the draft genome sequences of two Xanthomonas euvesicatoria strains from the Balkan Peninsula, which were isolated from symptomatic pepper plants. The availability of these genome sequences will facilitate the development of modern genotyping assays for X. euvesicatoria strains and to define targets for resistance breeding.

## GENOME ANNOUNCEMENT

First reported in the 20th century, bacterial spot has become an important disease affecting pepper and tomato production in the Balkan Peninsula (1–3). The causal agents of bacterial spot are included in the A2 list of pathogens of the European and Mediterranean Plant Protection Organization (http://www.eppo.int/QUARANTINE/quarantine.htm). Taxonomy and diversity of bacterial spot agents have been studied by phenotypic, biochemical, and DNA-based methods (4). When we applied these methods to Xanthomonas euvesicatoria strains isolated on the Balkan Peninsula (3, 5–9), we realized that these markers are not suitable to measure most population genetic parameters. A precise molecular typing tool for identification and differentiation is necessary for monitoring populations infecting pepper and tomato plants in order to develop strategies to control the disease in fields. To develop molecular markers of diversity, we decided to sequence the genomes of two X. euvesicatoria strains from the Balkan Peninsula.

Strain 66b, isolated from Capsicum annuum cv. Kambi in Bulgaria in 2012, and strain 83M, isolated from C. annuum cv. Kurtovska kapija in Macedonia in 2013, were chosen as representative X. euvesicatoria strains from the Balkan Peninsula based on their pathogenic, physiologic, and genetic characteristics (9). Their genomes were sequenced using the Illumina Hi-Seq2500 platform (Fasteris SA, Switzerland). The shotgun sequencing yielded 2,133,183 100-bp paired-end reads (533 Mb) for strain 66b and 1,605,465 paired-end reads (401 Mb) for strain 83M, with insert sizes ranging from of 250 bp to 1.5 kb. Draft genome sequences were assembled using the Edena algorithm v3.131028 (10), yielding 333 contigs larger than 500 bp (*N*_50_ = 28,793 bp) for strain 66b and 286 contigs (*N*_50_ = 31,385 bp) for strain 83M. Contigs were annotated with GeneMarkS+ release 2.9 (revision 452131) (11), as implemented in the NCBI Prokaryotic Genome Annotation Pipeline (http://www.ncbi.nlm.nih.gov/genome/annotation_prok/), predicting a total of 4,726 genes for strain 66b and 4,495 genes for strain 83M.

These genomic resources will aid in the development of modern molecular markers to monitor epidemics and to study the dynamics of Xanthomonas populations (12–16). The genome sequence was mined for the type III effector repertoires of the two strains from the Balkan Peninsula, using the Xanthomonas resource (http://www.xanthomonas.org). Interestingly, comparison with the completely sequenced strain 85-10, which was isolated in Florida in 1985 (17), revealed some unique effectors that are present or absent in one or the other strain (e.g., AvrBs1, AvrBs3, XopE3, XopG, XopH, XopAF, and XopAQ). Knowledge about presence and allelic diversity of type III effectors will help to define targets for developing resistance strategies (18).

### Nucleotide sequence accession numbers.

These whole-genome shotgun projects have been deposited at DDBJ/EMBL/GenBank under the accession numbers JSZG00000000 (strain 66b) and JSZH00000000 (strain 83M). The versions described in this paper are the first versions, JSZG01000000 and JSZH01000000.
